# Antitumour Effects of Isocurcumenol Isolated from *Curcuma zedoaria* Rhizomes on Human and Murine Cancer Cells

**DOI:** 10.1155/2011/253962

**Published:** 2011-02-15

**Authors:** S. Lakshmi, G. Padmaja, P. Remani

**Affiliations:** ^1^Division of Cancer Research, Regional Cancer Centre, Thiruvananthapuram, Kerala 695011, India; ^2^Division of Crop Utilisation and Biotechnology, Central Tuber Crops Research Institute, Thiruvananthapuram, Kerala 695011, India

## Abstract

*Curcuma zedoaria *belonging to the family Zingiberaceae has been used in the traditional system of medicine in India and Southwest Asia in treating many human ailments and is found to possess many biological activities. The rationale of the present study was to isolate, identify, and characterize antitumour principles from the rhizomes of *Curcuma zedoaria, *to assess its cytotoxic effects on human and murine cancer cells, to determine its apoptosis inducing capacity in cancer cells, and to evaluate its tumour reducing properties in *in vivo* mice models. Isocurcumenol was characterized as the active compound by spectroscopy and was found to inhibit the proliferation of cancer cells without inducing significant toxicity to the normal cells. Fluorescent staining exhibited the morphological features of apoptosis in the compound-treated cancer cells. *In vivo* tumour reduction studies revealed that a dose of 35.7 mg/kg body weight significantly reduced the ascitic tumour in DLA-challenged mice and increased the lifespan with respect to untreated control mice.

## 1. Introduction

Plants have been a source of medicine for thousands of years, and phytochemicals continue to play an essential role in medicine [1]. Several of the current chemotherapeutic drugs like vinblastine, methotrexate, taxol, and so forth, were first identified in plants. In developing countries, the practice of medicine still relies heavily on plant and herbal extracts for the treatment of human ailments. Dietary agents consist of a wide variety of biologically active compounds that are ubiquitous in plants, and many of them have been used as traditional medicines [2–4]. Some phytochemicals derived in spices and herbs as well as other plants possess substantial cancer preventive properties [5–10]. 

Plants of ginger family (Zingiberaceae) have been frequently and widely used as spices and in traditional systems of medicine practiced in many Asian countries, and their medicinal functions have been broadly discussed and accepted in many traditional recipes [11]. The Zingiberaceae plants contain a number of volatile and essential oils including terpenoids, phenylpropanoids, flavonoids, and sesquiterpenes, which has reported antitumour activity [12–14]. As plants of Zingiberaceae family are considered safe for human consumption, these species are excellent candidates for development of novel chemotherapeutics [15]. Traditionally, curcuma drugs called “Ukon” and “Gajutsu” in Japanese have been used in oketsu syndromes (caused by the obstruction of blood circulation) in Chinese medicine [16]. Various antineoplastic compounds have also been isolated from curcuma species [17–21].

*Curcuma zedoaria*, a medicinal tuber plant belonging to this family, is a close relative of *Curcuma longa* from which the curcumin is isolated. Various parts of *Curcuma zedoaria* are used in Ayurveda and other folk and tribal system of medicines [22]. *Curcuma zedoaria* is a constituent of a wide variety of ayurvedic preparations like Dasamularishtam, Valiya Rasnadi Kashayam, and so forth. The rhizome is used for curing stomach diseases, toothache, blood stagnation, leucoderma, tuberculosis, enlargement of spleen, and for promoting menstruation in traditional medicine in Asia [23]. Anti-inflammatory activity [24, 25], antifungal activity [26], antiulcer activity [27, 28], antimicrobial effect [13, 28, 29], hepatoprotective activity [30, 31], antiamoebic effect [32], of this plant rhizome have been reported. The *Curcuma zedoaria* rhizome is termed Ezhu in Chinese and is extensively used in traditional Chinese medicine to treat various ovarian and cervical cancers. Curdione isolated from this plant has been found to inhibit prostaglandin E2 production and cyclooxygenase-2 expression, both of which are implicated in inflammation and carcinogenic process [33].

Some novel compounds like curuminoids have been isolated from *Curcuma zedoaria* which has inhibitory activity against Ovarian cancer cell lines (OVCAR-3) [34]. Elemene isolated from *Curcuma zedoaria* rhizome has been found to have substantial antitumour activity against promyelocytic leukemic HL-60 cells [14]. Curcumin and curcumenol are reported to inhibit the growth of S-180 sarcoma cells and mouse cervical U-14 cells [13]. The rhizomes are reported to contain several sesquiterpenoid compounds [35, 36]. CZ-1-III, the polysaccharide fractions from *Curcuma zedoaria*, was found to decrease the tumor size sarcoma 180 of mouse and prevents chromosomal mutation [37]. Macrophage-stimulating activity of *Curcuma zedoaria* and its possibility of being used as a biological response modifier have been reported [38]. Essential oil isolated from *Curcuma wenyujin,* a close relative of *Curcuma zedoaria,* was found to induce apoptosis and inhibit the proliferation of human hepatoma and cervical cancer cells [39]. In the present study, attempts were made to isolate and characterize the active principles from the rhizomes of *Curcuma zedoaria* collected from Thiruvananthapuram district, Kerala, and to evaluate its cytotoxicity and antiproliferative activity on human lung, leukemic, nasopharyngeal, and murine cancer cell lines.

## 2. Experimental

### 2.1. Chemicals and Reagents

(3-(4,5-dimethylthiazol-2-yl)-2,5 diphenyltetrazolium bromide) (MTT), foetal bovine serum (FBS), acridine orange, ethidium bromide, Sigma-Aldrich, St. Louis, MO, USA; Dulbecco's Modified Eagle's Medium (DMEM), Rosewell Park Memorial Institute (RPMI), Hoechst 33342, Invitrogen; Cyclophosphamide Dabur Pharmaceuticals Ltd. All the chemicals used were of analytical grade.

### 2.2. Isolation and Purification of the Active Principles

The rhizomes of *Curcuma zedoaria *were collected from Central Tuber Crops Research Institute, Thiruvananthapuram, authenticated by the taxonomist, and a voucher specimen 41622 (TBGT) has been kept in the herbarium of Tropical Botanical Garden Research Institute, Thiruvananthapuram. As the preliminary studies conducted in our lab showed the potency of the petroleum ether fraction isolated from *C. zedoaria*, steps were taken to purify and characterize the antitumour principles from this fraction. The shade dried rhizomes were powdered (900 g) and were extracted with petroleum ether using a soxhlet apparatus for 48 hours. The yield of the extract obtained from *C. zedoaria* was 20.3 g. The petroleum ether extract isolated was made completely free of the solvent traces and mixed with cold diethyl ether and prewarmed silica. Suction was given to completely evaporate off the solvent. The contents obtained were poured in a Petri plate and kept in a hot air oven set at 50°C. After mixing it thoroughly, 10 grams of the petroleum ether extract were passed through columns packed with silica gel (100–200) mesh size set in Petroleum ether. Elution was done using the solvents 100% Petroleum ether, different ratios of Petroleum ether: ethyl acetate, ethyl acetate, different ratios of Ethyl acetate: methanol, and 100% methanol in the order of increasing polarity. Finally the column was washed with methanol alone. The different eluted fractions were collected and concentrated using a rotavapour.

### 2.3. Chemical Composition of the Active Petroleum Ether Fraction of *Curcuma zedoaria* by GC-MS

The compounds present in the active Petroleum ether fraction were identified by Gas Chromatography Mass Spectrophotometry (GC-MS) (GC-MS Shimadzu QP 2010). The specifications were the following: Column DB-5, injection temperature: 200°C, Interphase temperature: 200°C, ion source temperature: 200°C, The column temperatures were programmed from 50°C to 280°C at the rate of 100°C/min.

### 2.4. Thin Layer Chromatography

After concentrating the samples, thin layer chromatography (TLC) of the different samples collected was done. Samples which gave similar spots on TLC were pooled. Petroleum ether was added to the pooled samples and kept for crystallization.

### 2.5. Identification of the Active Fractions by Cytotoxicity Assay

The individual pooled fractions were subjected to MTT assay on DLA and A549 cancer cells to determine the active fraction among the various pooled fractions.

### 2.6. Characterization of the Active Compound

The dried crystals obtained were used for estimating mass spectra (MS) and infrared (IR) spectra. Mass spectrums were recorded on Agilent Technologies 1200 series Mass Spectrometer (Chem station software). IR spectrums were recorded on Perkin-Elmer spectrum 100-FTIR instrument (UK). It can identify unknown materials and can determine the quality or consistency of a sample and the amount of components in a mixture.

### 2.7. Cell Lines and Culture Conditions

Daltons Lymphoma Ascites (DLAs) cells were maintained in the peritoneal cavity of Balb/c mice (6–8 weeks old). Lung Carcinoma (A549), nasopharyngeal carcinoma (KB), leukemic (K562), Chicken embryo fibroblasts (normal primary cell lines) were maintained in Dulbecco's Modified Eagle's Medium (DMEM) supplemented with 10% Foetal Bovine Serum (FBS) in a 5% CO_2_ incubator at 37°C supplemented with penicillin (100 U/mL) and streptomycin (100 *μ*g/mL).

### 2.8. Isolation of Lymphocytes from Whole Blood

Three ml of blood were taken from normal healthy individuals and collected in heparinised test tube. Five ml of Phosphate Buffered Saline (PBS) were added and mixed well. Two ml of ficoll hypaque solution were taken and carefully layered blood PBS mixture on to the ficoll hypaque solution. It was centrifuged at 2000 rpm for 30 minutes. The opaque interface containing mononuclear cells was collected, mixed with PBS, and centrifuged at 1500 rpm for 10 minutes, and supernatant was discarded. The centrifugation was repeated thrice, and the lymphocyte pellet was resuspended in PBS.

### 2.9. Detection of Cell Viability by MTT Assay

The active compound was dissolved in Dimethyl Sulfoxide (DMSO) and used for MTT assay. Briefly 5 × 10^3^ cells were cultured and incubated for 24, 48, and 72 hours. At the end of incubation period, MTT was added to all the wells and incubated in dark for 2 hours at 37°C. Then lysis solution (20% Sodium Dodecyl Sulphate (SDS) in 50% dimethyl formamide (DMF)) was added and further incubated for 4 hours in dark. After the incubation, the optical density was assessed at 570 nm using a multiwell plate reader [40]. 5-Fluorouracil was used as the positive control for DLA and A549 cells and doxorubicin for KB and K562 cells. The Inhibitory concentration required for 50% cytotoxicity (IC_50_) value was analysed using Easyplot software.

### 2.10. Analysis of Apoptotic Features by Acridine Orange-Ethidium Bromide and Hoechst Staining

The morphological features of apoptosis induced by the active compound were evaluated by Acridine Orange-Ethidium Bromide dual (AO/EtBr) staining [41] and Hoechst staining [42]. Briefly, cells were seeded in a 96-well plate at a density of 5 × 10^5^ cells and then treated with different concentrations of the compound for 48 hours. After washing once with phosphate buffered saline (PBS), the cells were stained with 100 *μ*L of a mixture (1 : 1) of acridine orange-ethidium bromide (4 *μ*g/mL) solutions. The cells were immediately washed with PBS and observed under fluorescence microscopy at 450–490 nm. The number of cells manifesting morphological features of apoptosis such as nuclear fragmentation and chromatin condensation were evaluated. 

For Hoechst staining, the cells were seeded in a 96-well plate at a density of 5 × 10^5^ cells and then treated with different concentrations of the compound for 48 hours. After washing once with PBS, the cells were stained with 100 *μ*L of Hoechst 33342 (10 mg/mL stock) and incubated at room temperature for 5 minutes. Stained cells were imaged by fluorescence microscopy at 350–460 nm. The number of cells manifesting chromatin condensation was counted as a function of total number of cells present in the field. 

### 2.11. Acute and Subacute Toxicity Studies in Mice Models

Acute toxicity studies were carried out in Balb/C mice (28–30 g) (6 animals per group) by a single intraperitoneal injection of different concentrations of the active purified fraction isolated from *Curcuma zedoaria *(CZ-PF). The LD_50_ value (lethal dose for 50% animals to be killed) was assessed and calculated. Subacute toxicity studies were done by intraperitoneal injection of different sublethal doses of LD_50_ value once a day for 14 days continuously. Animals treated with normal saline were taken as negative control and those treated with the standard drug, cyclophosphamide (CYP), were taken as positive control. After 14 days, the animals were sacrificed, and the blood was collected to estimate various liver and kidney function tests such as (Serum Glutamate Ortho-Transferase (SGOT), Serum Glutamine Pyruvate Transaminase (SGPT), Alkaline Phosphatase (ALKP), creatinine, and urea) to assess any toxicity induced by the active fraction.

### 2.12. Ascitic Tumour Reduction Studies in Mice Models

Balb/c mice (28–30 g) (6 animals per group) were treated with the active fraction alternatively for 21 days one day post-DLA ascitic tumour transplantation. After the treatment period, the ascitic fluid was aspirated and calculated using the formula *V*3 = (*V*1 + *V*2) − *V*2, where *V*3 is the total ascetic fluid volume, *V*1 is the volume of the ascetic fluid obtained from the peritoneum, and *V*2 is the volume of the added saline [43]. Increment in lifespan (ILS) was assessed according to the formula % ILS = (*T* − *C*)/*C* × 100, where *T* represents median survival time of the treated animals and *C* represents the median survival time of the control group.

### 2.13. Statistical Analysis

All the *in vitro* experiments were done in triplicates and for the *in vivo* experiments, six animals per group were used. Results are expressed as mean ± standard deviation. Statistical analysis was done by means of one-way Anova followed by Turkey-Kramer multiple comparison test using Graphpad Insta software. *P* value <.05 was considered statistically significant.

## 3. Results

### 3.1. GC-MS Analysis of the Active Fraction

**Figure 1:**
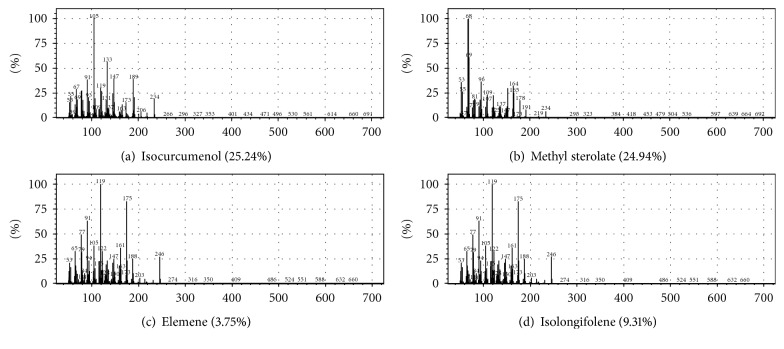
Mass spectra of the four major compounds present in Petroleum ether fraction of *Curcuma zedoaria.* (a) Isocurcumenol. (b) Methyl sterolate. (c) Elemene. (d) Isolongifolene.

The phytoconstituents present in the active Petroleum ether extract of *Curcuma zedoaria* are given in Table 1. The GC-MS analysis revealed that isocurcumenol (25.24%), methyl sterolate (24.94%), isolongifolene (9%), and elemene (3%) are the major compounds present in the Petroleum ether fraction. The mass spectra of the compounds are given in Figure 1.

### 3.2. Cytotoxicity of the Active Fractions Isolated from * Curcuma zedoaria*

The cell viability assay by MTT revealed that 60 : 40 Petroleum ether: ethyl acetate fraction is the active fraction with antiproliferative effects against DLA and A549 cancer cells when treated for 24 and 48 hours. The IC_50_ values are given in Table 2. 

### 3.3. MS Spectral Data of the Active Compound Isolated from *Curcuma zedoaria*

**Figure 2:**
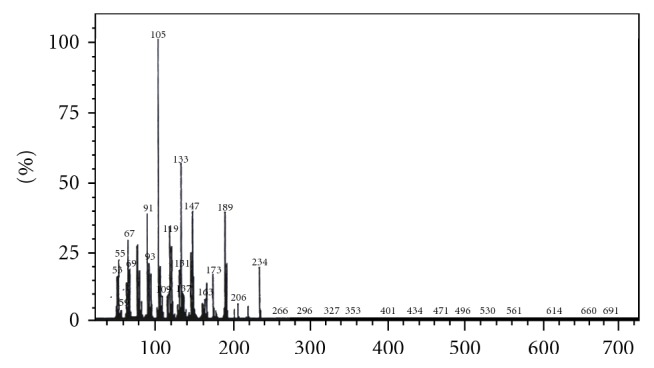
MS Spectrum of the active compound isolated from *Curcuma zedoaria. *

MS (m/z), that is, mass/charge ratio is 51, 55, 67, 69, 91, 93, 105, 109, 119, 131, 133, 137, 147 163, 173, 189, 206, 234. The mass fragmentation pattern suggests the molecular weight of the active compound isolated from *Curcuma zedoaria *to be 234 (see Figure 2).

### 3.4. IR Spectral Data of the Active Compound Isolated from *Curcuma zedoaria*

**Figure 3:**
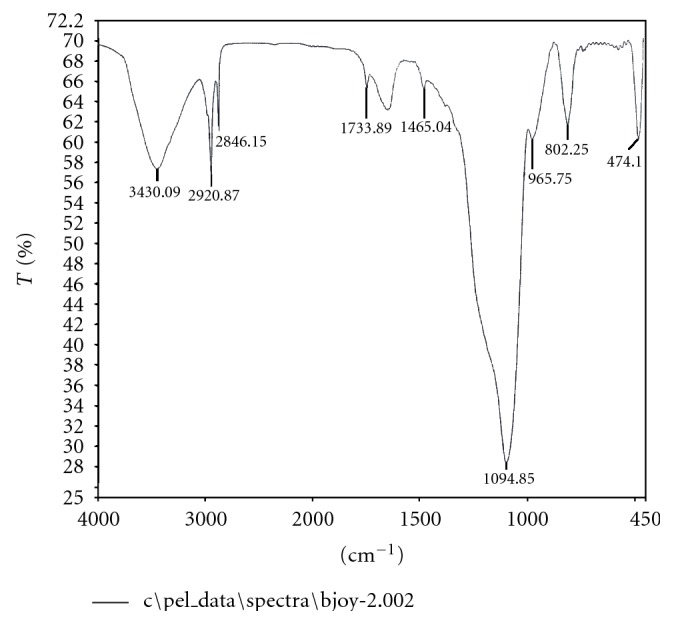
IR Spectrum of the active compound isolated from *Curcuma zedoaria. *

IR (cm^−1^) – 474 (w), 802.25 (w), 1094.85 (s), 1465 (w), 1733.89 (w), 2846 (w), 2920 (w), 3430 (b) (see Figure 3).

### 3.5. Structural Configuration for the Active Compound Isolated from *Curcuma zedoaria* from the Spectral Data

**Figure 4:**
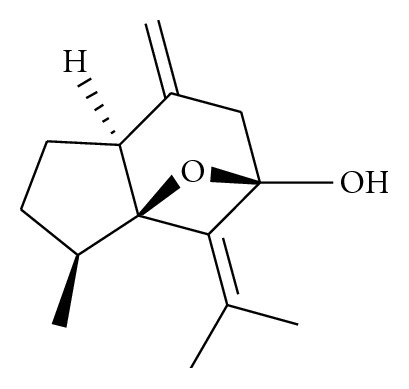
Structure of isocurcumenol purified from *Curcuma zedoaria. *

From the Petroleum ether: ethyl acetate (60 : 40) of *C. zedoaria*, a pure yellow coloured semisolid oil like compound was isolated. The melting point of the compound isolated was estimated to be 144°C experimentally. The active compound crystallized was assigned as isocurcumenol. The structure was confirmed by comparing the spectral values, molecular mass, and the melting point with those reported in the literature [58]. The yield of isocurcumenol isolated from 10 grams of the Petroleum ether extract was 30.7 milligrams. The fraction containing the pure compound isolated from *C. zedoaria* was developed in 50 : 50 Petroleum ether: Ethyl acetate and developed by spraying using 10% sulphuric acid in methanol. Phytochemical analysis revealed that the pure compound isolated was of sesquiterpenoid class (see Figure 4).

### 3.6. Inhibition of Cancer Cell Proliferation by Isocurcumenol

**Figure 5:**
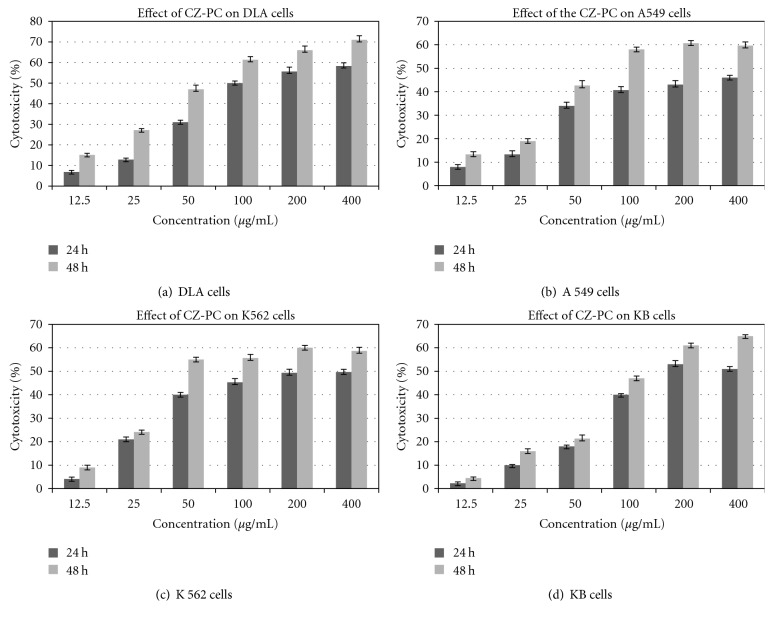
Comparison of the % of cytotoxicity between the cancer cells after 24 and 48 hours of incubation with isocurcumenol (CZ-PC)—*Curcuma zedoaria* pure compound. (a) DLA, (b) A549, (c) K 562, and (d) KB cells.

**Figure 6:**
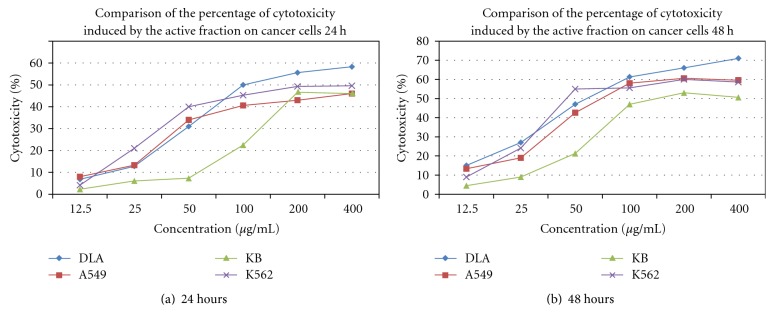
Comparison of the % of cytotoxicity of DLA, A549, KB, and K562 cells after treatment with isocurcumenol for 24 (a) and 48 (b) hours.

**Figure 7:**
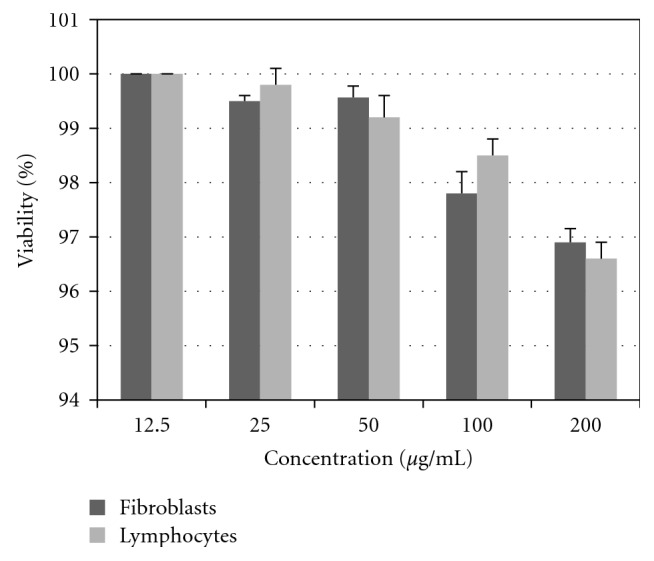
Percentage viability after treatment with isocurcumenol on normal lymphocytes and chicken embryo fibroblast cells—72 hours.

The cell viability assay revealed the potent cytotoxic activity of CZ-PC (*Curcuma zedoaria* Purified compound), isocurcumenol on various cancer cells (Figures 5(a), 5(b), 5(c), and 5(d)). There was a concentration and time-dependent increase in the percentage of cytotoxicity in DLA, A549, K-562 and KB cells. All values obtained were highly significant compared to the untreated control cells (*P* < .001). 20 *μ*g/mL of 5-fluorouracil inhibited 83% and 71% of cell proliferation in A549 and DLA cells, respectively, whereas 10 *μ*g/mL of Doxorubicin inhibited 59% and 78% of cell proliferation in K562, and KB cells, respectively. The comparisons between the percentage of cytotoxicity induced by isocurcumenol (CZ-PC) in the different cancer cell lines are given as Figures 6(a) and 6(b). DLA, A549, and K562 cells were more sensitive to CZ-PF than KB cells. The IC_50_ values were also determined after 24 and 48 hours of treatment (Table 3). On the contrary, isocurcumenol did not exhibit any significant toxicity on normal chicken embryo fibroblast cells and lymphocytes (Figure 7). 

### 3.7. Induction of Apoptosis by CZ-PF

**Figure 8:**
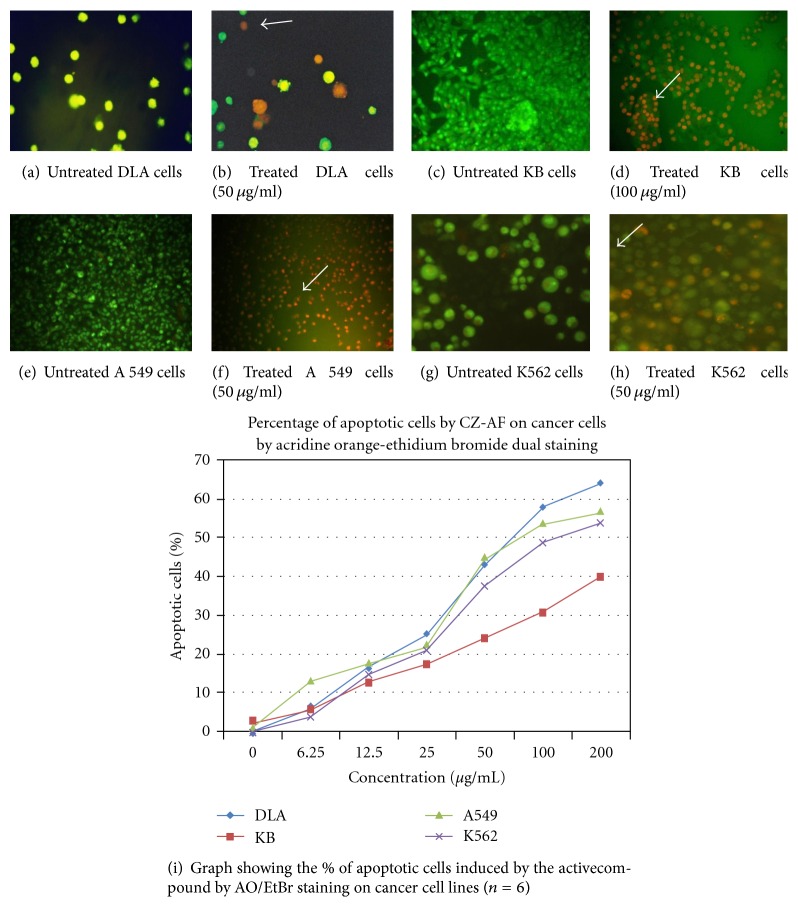
AO/Et Br staining in DLA cells treated with isocurcumenol (a and b). Untreated and 50 *μ*g/mL treated DLA cells (c and d). Untreated and 100 *μ*g/mL treated KB cells (e and f). Untreated and 50 *μ*g/mL treated A 549 cells (g and h). Untreated and 50 *μ*g/mL treated K-562 cells. (i) Graph showing the % of apoptotic cells induced by treatment with isocurcumenol by AO/EtBr staining on cancer cell lines. (↑) shows the apoptotic cells.

**Figure 9:**
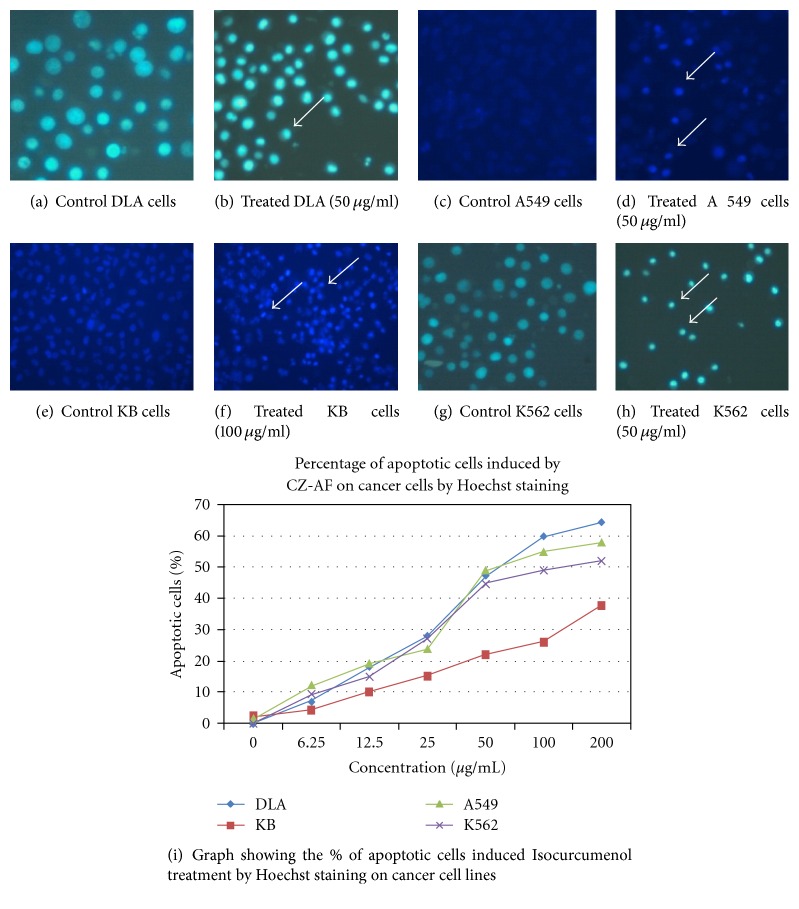
Hoechst staining in DLA cells treated with isocurcumenol (a and b). Untreated and 50 *μ*g/ml treated DLA cells (c and d). Untreated and 100 *μ*g/ml treated KB cells (e and f). Untreated and 50 *μ*g/ml treated A 549 cells (g and h). Untreated and 50 *μ*g/ml treated K562 cells. (i) Graph showing the % of apoptotic cells induced by the treatment with isocurcumenol by Hoechst staining on cancer cell lines. (↑) shows the apoptotic cells with chromatin condensation.

Dual staining by acridine orange ethidium bromide revealed the characteristic features of apoptosis like chromatin condensation, nuclear fragmentation, and membrane blebbing after 48 hours of treatment with CZ-PF in DLA, A 549, K562, and KB cells (Figure 8). Hoechst staining revealed a greater intensity of blue colour in all the treated cells compared to control cells, which is due to the condensed chromatin, the characteristic feature of apoptosis (Figure 9). There was a concentration-dependent increase in the percentage of apoptotic cells after 48 hours of incubation. There was a significant increase (*P* < .001) in the percentage of apoptotic cells in the DLA, A 549, K562, and KB cells at concentrations of 25, 50, 100, and 200 *μ*g/mL.

### 3.8. Toxicity Profile in Mice Models

**Figure 10:**
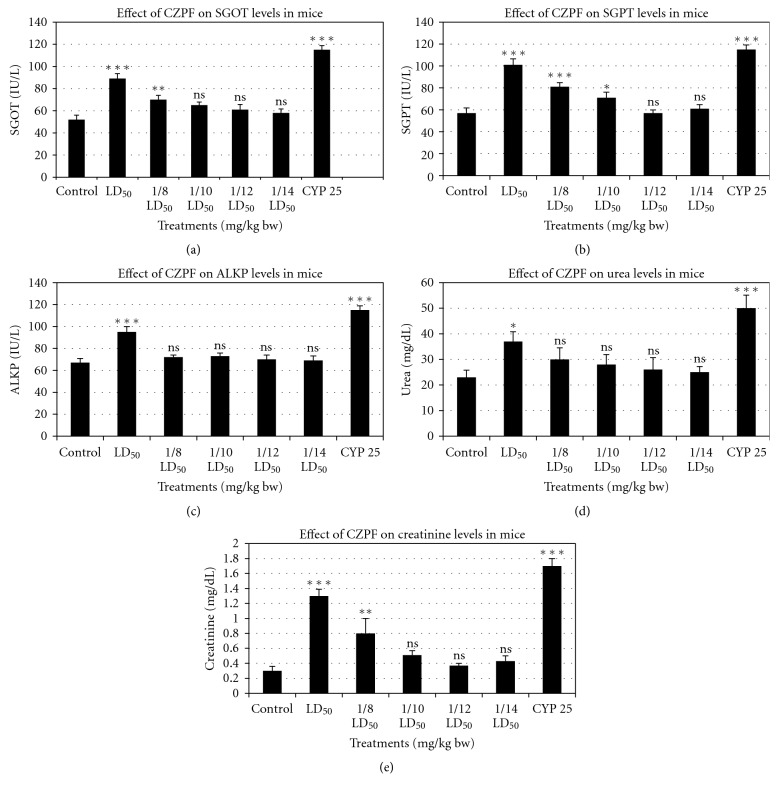
Graphs showing the relative toxicity of different concentrations of CZ-PF and cyclophosphamide on biochemical parameters in animal models. (a) SGOT. (b) SGPT. (c) ALKP. (d) Creatinine. (e) Urea (∗*P* < .05, ∗∗*P* < .01, ∗∗∗*P* < .001, ^ns^ *P* > .05).

From the acute toxicity studies, it was found that a dose of 500 mg/kg bw of CZ-PF is the LD_50_ value. Taking LD_50_ values as the reference value, 1/8th, 1/10th, 1/12th, and 1/14th of LD_50_ values were employed for the further subacute toxicity studies (Figure 10). The results suggested that the purified fraction in low doses manifested no toxic symptoms while very high doses marked toxic as well as depressant activities. Usually the liver and kidney enzymes tend to deviate from their normal range to higher values upon toxicity. Treatment of CZPF (1/12th and 1/14th) of LD_50_ values, that is, 41.6 and 35.7 mg/kg bw, did not alter significantly the levels of SGOT, SGPT, and ALKP levels (liver enzymes) (Figures 10(a), 10(b), and 10(c)) as well as those of creatinine and urea (kidney enzymes) (Figures 10(d) and 10(e)) (*P* > .05). However animals treated with LD_50_ doses of CZPF, that is, 500 mg/kg bw exhibited significant toxicity on the liver and kidney enzyme levels (*P* < .001), but lower than that of the animals treated with cyclophosphamide. Animals treated with 1/8th of LD_50_ values of CZPF, that is, 62.5 mg/kg bw, exhibited marginal toxicity on SGOT and ALKP levels and significant toxicity on SGPT levels. The same dose of CZPF induced no significant toxicity on urea levels, whereas the same dose induced marginal toxicity in the creatinine levels. These results emphasize the nontoxicity of the purified fractions of CZPF at concentrations of (1/12th and 1/14th) of LD_50_ values, 41.6 and 35.7 mg/kg bw, respectively. 

### 3.9. Ascitic Tumour Reduction in Mice Challenged with DLA Cells

**Figure 11:**
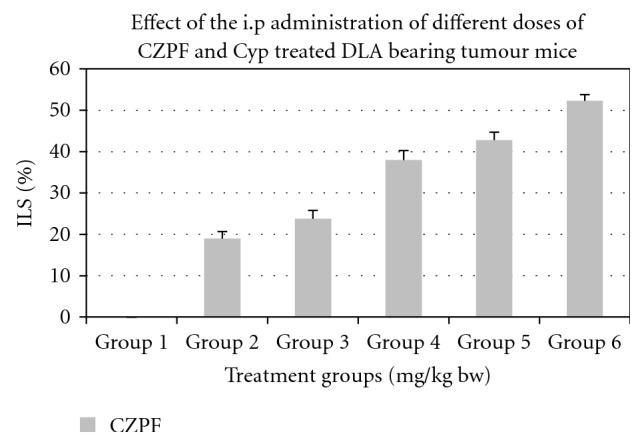
Effect of the i.p administration of different doses of CZPF and cyclophosphamide on the life span of DLA bearing mice.

**Figure 12:**
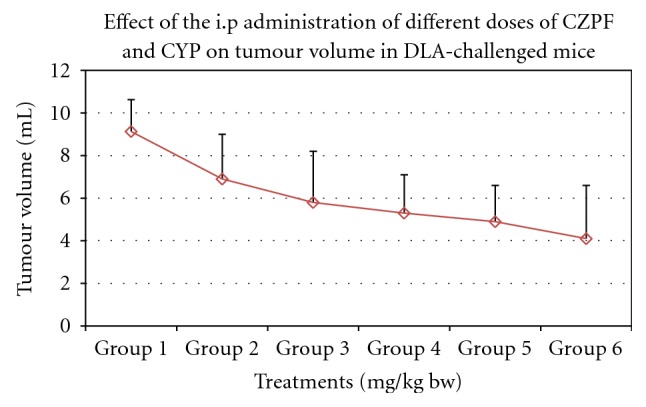
Effect of the i.p administration of different doses of CZPF and CYP on tumour volumes in DLA-challenged mice.

6 groups of animals with specific treatment schedule were assigned. Treatment with 1/12th and 1/14th doses of LD_50_ values of CZPF showed that the tumour volume regressed in size from 9.13 ± 2.1 in control to 5.3 ± 1.7 and 4.8 ± 1.8, respectively, and subsequently increased the lifespan of mice to 38% and 42.8%, respectively. These results revealed that the optimum dose for the maximum antitumour activity of CZPF was 35.7 mg/kg bw, that is, 1/14th of LD_50_ value. In corresponding animals treated with CYP, the tumour volume regression was observed to be a value very near to that of those treated with 35.7 mg/kg bw of CZPF.  Table 4 illustrates the effects of the i.p administration of different doses of CZPF and cyclophosphamide on the lifespan of DLA bearing mice. Figures 11 and 12 illustrate the effects of the i.p administration of different doses of CZPF in comparison with CYP on the life span and tumour volumes in DLA-challenged mice.

## 4. Discussion

The Zingiberaceae plants are well reported to possess many bioactive compounds and are known to induce apoptosis in cancer cells. 6-gingerol isolated from *Zingiber offinalis* was found to inhibit the proliferation of rat colonic adenocarcinoma cells and angiogenesis [44]. Zerumbone isolated from the rhizomes of *Zingiber zerumbet* is one of the most promising chemopreventive agents against colon and skin cancer cells [45] and is shown to induce apoptosis in various tumour cells [46, 47]. This compound is also found to induce antiproliferative effects in the breast carcinoma cells [48], exerts apoptosis through Caspase 3 activation in He La cells [49], and modulates the Bax/Bcl-2 ratio in liver cancer cells [50].

Various antineoplastic compounds have been isolated from the Curcuma species [20]. Curcumin isolated from *Curcuma longa* has been shown to suppress the proliferation of a wide variety of tumour cells through downregulation of antiapoptotic gene products, activation of caspases, and induction of tumour suppressor genes such as p53 [51–53]. Curdione isolated from *Curcuma aromatica*, a close relative of *Curcuma zedoaria,* was found to show inhibitory effects on Caco-2 cells [54]. Furanodiene is one of primary anticancer active components in the essential oil of *Curcuma wenyujin*, very effective agent against uterine cervix cancer and has protection effect on the immune function [55]. Curcuminoids isolated from *C. zedoaria *rhizomes were demonstrated to be cytotoxic against human ovarian cancer OVCAR-3 cells [34]. The antitumor effect of the partially purified polysaccharide from *C. zedoaria* was studied in mice transplanted with sarcoma 180 cells, and the results suggested that CZ-1-III, the polysaccharide fraction from *C. zedoaria*, decreased the tumor size of mouse and prevented the chromosomal mutation [37]. An essential oil isolated from *C. zedoaria* was found to possess inhibitory effects against human promyelocytic leukemia HL-60 cells. The GC-MS results demonstrated the presence of epicurzerenone and curdione [13]. Our study reports the significant nontoxic nature and antitumour effects of isocurcumenol, a sesquiterpenoid compound isolated from *Curcuma zedoaria *on the human lung, nasopharyngeal, and leukemic cells as well as the murine lymphoma cells.

Shibuya et al. [56] reported that furanogermenone, curcumenol, and dehydrocurdione were the major oil components of species of *C. zedoaria* native to China, Taiwan, and Japan [56]. Curzerenone has also been reported as major oil component of the *C. zedoaria* rhizomes [36, 57–59]. Our studies on the plant rhizomes native to Kerala showed isocurcumenol and methyl sterolate as the major compounds. The present finding could indicate the existence of different chemotypes and regional variation for *C. zedoaria*.

Earlier reports show that phytochemical investigations carried out in the Curcuma species, namely*, Curcuma ochrorhiza* and *Curcuma heyneana* of the Malaysian origin, have resulted in the isolation of six sesquiterpenes including isocurcumenol [60]. Isocurcumenol has already been isolated from *Curcuma zedoaria* of the Chinese origin [36, 58], and we could isolate the same compound from rhizomes of Indian origin too.

The curcuminoids isolated from *Curcuma zedoaria* were reported to inhibit the growth of ovarian carcinoma (OVCAR-3), leukemic (HL-60), S-180 sarcoma, and mouse cervical U-14 cells [13, 14, 34]. In the present study, among the four cancer cell lines tested, isocurcumenol induced significant antiproliferative effects in the inhibition of proliferation of DLA, A 549, K562, and KB cells.

Intraperitoneal administration of the purified fractions from the plant rhizomes exhibited differential toxicity profile in mice at different doses. The results showed that the animals could tolerate a reasonably high dose of CZ-PF. The treatment of the purified fractions at sublethal doses did not decrease the food and water intake as well as the body weight. Estimation of the liver function tests of SGOT, SGPT, and ALKP is mostly used for measuring hepatocellular injury. The microscopic analysis of the target organs, liver, spleen and, kidney, did not show significant changes in architecture and texture in animals treated with 1/12th and 1/14th of sublethal doses when compared with the control group. The increase in SGOT and SGPT in serum at very high doses may be due to hepatocellular necrosis, which causes increase in the permeability of the cell membrane resulting in release of transaminases into the blood stream. Also there was not much toxicity in the kidney function tests as reflected from the levels of urea and creatinine at these doses. The normal levels of blood urea and creatinine indicated that the purified fractions at these doses did not interfere with the renal function and the renal integrity was preserved.

The reliable criteria for judging the value of any anticancer drug is prolongation of life span [61]. The purified fraction, CZ-PF, showed profound antitumour effects against DLA cells in mice models as evidenced by the increase in the lifespan and decrease in the ascitic tumour volume. From the results obtained, we can conclude that there was a good response of antitumour activity in *in vitro* cell culture experiments as well as *in vivo* antitumour studies. All the animals were apparently healthy indicating no side effects. The cell culture experiments showed remarkable activity in arresting the growth of tumour cell lines of different origin both in crude and purified forms. All these indicated that the plants possess a potential antitumour activity. The good antitumour responses observed were due to the potent sesquiterpenoid compound isocurcumenol isolated from the purified fraction of *Curcuma zedoaria*. immunomodulation, Immunostimulation, effects on humoral immune response, anti-angioneogenesis activity in addition to cytotoxicity towards the tumour cells might be the probable mechanisms by which the reduction in tumour growth was achieved after treatment with CZ-PF. As CZ-PF is having good safety profile without much of toxic effects, it may be ideal candidate for prospective trials on more tumour cells. 

## 5. Conclusions

The compound isolated from the rhizomes of *Curcuma zedoaria, *characterized as isocurcumenol by the MS and IR spectra significantly inhibited the cell proliferation in human lung, leukemia, nasopharyngeal carcinoma and murine lymphoma cells. Acridine orange-Ethidium Bromide and Hoechst staining revealed the apoptosis inducing capacity of isocurcumenol. GC-MS profile of the Petroleum ether extract showed isocurcumenol, methyl sterolate, elemene, and Isolongifolene as the prominent chemical constituents. The* in vivo* studies suggested the non toxic nature of the compound at low doses and its antitumour effects in the ascitic tumour development comparable to the standard drug used to treat lymphoma, cyclophosphamide. The present study highlights the antitumour potential of isocurcumenol isolated from *Curcuma zedoaria* to be exploited further to be developed as a good antitumour agent.

## Figures and Tables

**Table 1:** GC-MS profile of the Petroleum ether fraction of *Curcuma zedoaria* shows the major phytoconstituents and their percentage.

Sl No:	Compound	Retention time (minutes)	Percentage (%)
1	Eucalyptol	9.786	2.04
2	Camphor	12.44	1.58
3	Beta-selinene	21.008	1.03
4	Azulene	21.225	0.87
5	4,5,6,6A-tetrahydro 2 [1H] pentalenone	23.752	14.49
6	Germacrone	25.783	1.87
7	Isocurcumenol	26.623	25.24
8	9-octadecynoic acid methyl ester	26.948	1.36
9	Dihydro neoclovene	27.018	3.48
10	Methyl sterolate	27.738	24.94
11	Isovelleral	27.886	5.92
12	Eremanthron	28.127	2.89
13	Elemene	28.883	3.75
14	Octahydroanthracene	31.650	1.25
15	Isolongifolene	31.961	9.31

**Table 2:** Cytotoxicity of the active fractions isolated from * Curcuma zedoaria. *

Cell lines	60 : 40 PE : EA CZ fraction IC_50_ values (*μ*g/mL)	
	24 h	48 h

DLA	95.5 ± 1.7	77.2 ± 3.1
A549	>400	73.2 ± 2.5

**Table 3:** IC_50 _ values on different cancer cells induced by treatment with CZ-PC (Isocurcumenol).

Cells	IC_50_ value (*μ*g/mL) (CZ-PC)	
	24 h	48 h

DLA	99.1 ± 1.7	75.3 ± 1.5
A549	>400	75.7 ± 1.3
KB	178.2 ± 0.4	142.2 ± 1.1
K562	>400	45.83 ± 2.4

**Table 4:** Effect of the i.p administration of the purified fraction of *Curcuma zedoaria *on the life span of DLA tumour bearing mice.

Treatments	No: of mice survived after tumour inoculation (days)			Mean survival time (MST) (days)	Increase in life span (%)
	15	25	35		

Group 1 (DLA Control)	6/6	0/6	0/6	21 ± 2.1	—
Group 2 CZPF 62.5 mg/kg bw	6/6	3/6	0/6	25 ± 1.7	19.0
Group 3 CZPF 50 mg/kg bw	6/6	4/6	0/6	26 ± 2.0	23.8
Group 4 CZPF 41.6 mg/kg bw	6/6	4/6	0/6	29 ± 2.3	38.0
Group 5 CZPF 35.7 mg/kg bw	6/6	5/6	0/6	30 ± 1.9	42.8
Group 6 CYP (25 mg/kg bw)	6/6	6/6	3/6	32 ± 1.5	52.3
